# Measuring Depression in Young Adults: Preliminary Development of an English Version of the Teate Depression Inventory

**DOI:** 10.3390/ijerph20156470

**Published:** 2023-07-28

**Authors:** Linda Ruan-Iu, Laura L. Pendergast, Pei-Chun Liao, Paul Jones, Nathaniel von der Embse, Marco Innamorati, Michela Balsamo

**Affiliations:** 1 Department of Psychological Studies in Education and Human Development, College of Education, Temple University, Philadelphia, PA 19122, USA; 2 Division of Child & Adolescent Psychiatry, Columbia University Irving Medical Center, New York, NY 10032, USA; 3 College of Education, University of South Florida, Tampa, FL 33620, USA; 4 Department of Human Sciences, European University of Rome, 00163 Roma, Italy; 5 Department of Psychological Sciences, Humanities and Territory, “G. d’Annunzio” University of Chieti-Pescara, 66100 Chieti, Italy

**Keywords:** depression, structural validity, concurrent validity, Tripartite Model, young adults

## Abstract

Depression is a common and debilitating condition that impacts individuals with various cultural backgrounds, medical conditions, and life circumstances. Thus, assessment tools need to be useful among different cultural groups. The 21-item Teate Depression Inventory (TDI) was developed in Italy, is designed to assess major depression, and focuses on cognitive and affective rather than somatic symptoms. This study aims to examine the factor structure and concurrent validity of the TDI English version among a non-clinical population in the United States. Participants included 398 adults (mean age 19.89 years, SD = 2.72, range: 18 to 46 years old) who completed the TDI and The Center for Epidemiologic Studies Depression Scale-Revised (CESD-R). The results supported a three-factor bifactor structure of the TDI (Positive Affect, Negative Affect, and Daily Functioning), which largely corresponds to the Tripartite Model of affective disorders. These findings support the use of TDI scores as measures of depressive symptoms among U.S. young adults, offering researchers and practitioners a brief and useful tool.

## 1. Introduction

Depression is a mental disorder that is pervasive worldwide [1]. More than 350 million people are affected by depression, making it one of the most common mental disorders [1,2]. Individuals with depression commonly exhibit affective (e.g., impairment of mood regulation, loss of interest), cognitive (e.g., diminished concentration) and somatic (e.g., decreased energy, significant weight change) symptoms [3]. The burden of depression is rising globally [4] and is often long-lasting [5,6,7]. Although depression often impacts individuals from a wide array of cultural backgrounds and individuals with chronic health conditions, the assessment of depression across diverse groups presents many challenges.

### 1.1. Assessment of Depression

A multifaceted approach (multimethod, multi-setting, multisource) is recommended as best practice for the assessment of emotional disorders, including depression [8]. Because depression involves internal states and internalizing symptoms, clinicians and researchers rely heavily on self-report scales alongside diagnostic tools (e.g., semi-structured interview) in the assessment of depression. Self-rating scales, in particular, are commonly used for screening depression and measuring symptom severity throughout the course of treatment. Thus, self-report rating scales are essential tools in the assessment of depression [9].

Several self-rating depression measures with strong psychometric properties exist. These may include the Patient Health Questionnaire (PHQ-9), the Center for Epidemiologic Studies Depression Scale-Revised (CESD-R), and the Hospital Anxiety and Depression Scale (HADS) [10,11,12]. These widely used measures are of high quality, and there is no evidence that any measure is superior to another [13,14].

Although self-report measures are highly utilized in the assessment of depression, these instruments can also present challenges. First, physical symptoms are often included in depression scales (e.g., PHQ-9, CESD-R), and those with physical health conditions (e.g., chronic pain, post-partum women) may exhibit physiological symptoms similar to patients with depression (e.g., sleep disturbance, fatigue, appetite problem) [15,16]. Hence, a heavy reliance on somatic symptoms may complicate the assessment of depression, creating a challenge for researchers and clinicians to accurately assess for depression among people with certain health conditions, thereby reducing measurement accuracy. Other measures that exclude somatic symptoms (e.g., HADS) may omit questions on suicidal thoughts, an important symptom of depression often required in self-reported measures used for screening and monitoring patients with depression [17]. Second, many scales are lengthy, which can be laborious for patients [18,19] and inconvenient for progress monitoring. Further, because depression is an international phenomenon, it is necessary for clinicians to have access to measures of depressive symptoms that function well in different cultural settings [20]. Finally, it is important for all psychological scales, including those measuring depression, to correspond with theory [21].

### 1.2. Tripartite Model

Given the substantial comorbidity of depression and anxiety, Clark and Watson proposed the Tripartite Model, which explains the overlap between depression and anxiety and provides a mechanism for differentiating them. According to this model, individuals with depression display low levels of Positive Affect (PA; e.g., interest, enthusiasm) and high levels of Negative Affect (NA; e.g., sadness, distress). However, depression is expected to be unrelated to physiological hyperarousal (PH; e.g., heart pounding, restlessness), which is associated more with anxiety [22]. This three-factor structure (i.e., positive affect, negative affect, and physiological hyperarousal) has been supported by many studies focused on a variety of populations [23,24,25]. The Tripartite Model is an emerging and increasingly well-studied theory of internalizing disorders [25,26], but relatively few measures correspond to this model and are also brief, culturally sensitive, and appropriate for individuals with varying health conditions.

### 1.3. Teate Depression Inventory

The Teate Depression Inventory (TDI) [9,20] is a newly developed, self-report depression scale that addresses some limitations of currently available measures [18,19]. The TDI has several advantages. First, the TDI is a brief measure with 21 items [27,28]. Second, the TDI focuses on cognitive and affective symptoms instead of somatic ones, allowing the scale to be more applicable to individuals with chronic health conditions and making it particularly useful for researchers comparing depression across individuals with and without chronic health conditions [28,29]. Third, there is a growing body of literature that has supported the Italian TDI’s psychometric properties in both clinical and non-clinical samples [27,28,29,30,31,32], including no evidence of bias due to item-trait interaction, good discriminant and convergent validity [30,31,33,34,35], criterion-related validity with the BDI-II [28,29], and excellent internal consistency across non-clinical and clinical samples [28,29,31,35]. Given previous findings supporting the reliability and validity of TDI scores in Italy [30,31,32,35,36,37,38,39], it may be fruitful to examine the utility of the TDI among countries outside of Italy.

The newly developed English TDI has the potential to be useful for a wide array of populations within the United States, including individuals with chronic health conditions. However, the structural validity of the TDI has not yet been examined using a factor analytic approach in the United States. Finally, although the items on the TDI were designed to assess major depression as specified by the latest editions of the DSM [3,40], they should be reflective of a theory of affective disorders. Although they appear to be well-aligned with the Tripartite Model, the structural correspondence has not yet been examined.

## 2. Materials and Methods

To date, TDI’s psychometric research has been primarily restricted to Italy; no research has examined the psychometric properties of TDI scores in the United States. However, the TDI was developed with the intent for the items to be relevant across cultures. The study aims to develop an English version of the TDI and to conduct a preliminary examination of the psychometric properties of TDI scores among a non-clinical, young-adult sample in the United States. Three hypotheses were tested: (1) a higher-order bifactor model would sufficiently account for the covariance of the TDI items, (2) the TDI’s structure would correspond with the Clark & Watson’s (1991) Tripartite Model—particularly with regard to the PA and NA factors—and (3) the TDI scores would be moderately correlated with the CESD-R, another well-established measure of depression.

### 2.1. Participants

The participants were 409 undergraduates (119 male, 283 females, three of another gender, four did not report a gender; M age = 19.89 years; SD = 2.72; range: 18 to 46 years old) at a large, urban university in the northeastern United States. A total of 65% identified as White/European American, 12% Asian American/Pacific Islander, 11% Black/African American, 5% Multi-Racial, 4% reported “other” race, 2% preferred not to respond, 1% identified as American Indian/Native American, and 8% identified as Hispanic/Latino. Sexual orientations were reported: 83% identified as heterosexual, 7% bisexual, 4% lesbian or gay, 4% preferred not to respond, 1% was questioning, and <1% reported “other” sexual orientation (majority identified as genderqueer). Among the 409 participants, 11 participants had missing data. Due to minimal missingness (<3%), these 11 participants were removed, resulting in a final sample size of 398 participants.

### 2.2. Measures

Teate Depression Inventory (TDI). The original 21-item TDI [27] was translated into English following the standard procedure of forward and back translation [41]. First, the original items on the Italian version were translated into English by a bilingual psychologist. Next, a bilingual translator with a strong background in mental health translated the English version back into Italian. Then, the scale developers (who are bilingual) compared the back-translated version with the original version. Finally, the scale developers, alongside an expert panel, evaluated the English version.

The TDI uses a 5-point Likert-type scale (1 = “never”, 2 = “rarely”, 3 = “sometimes”, 4 = “often”, 5 = “always”), and respondents rate the frequency of depressive symptoms (α = 0.95). Higher TDI scores indicate more severe depressive symptoms.

The Center for Epidemiologic Studies Depression Scale-Revised (CESD-R) [11]. The CESD-R is a 20-item self-report depression rating scale (α = 0.95) that uses a 5-point Likert-type scale (0 = “Not at all or Less than one day”, 1 = “1–2 days”, 2 = “3–4 days”, 3 = “5–7 days”, 4 = “Nearly every day for 2 weeks”) and consists of two subscales (Functional Impairment and Negative Mood). One item involving suicidal ideation was removed because our anonymous data-collection process would have prevented follow-up. Previous research supports the structural, convergent, and divergent validity of CESD-R scores [35]. Using the CESD-R scoring criteria as reference, approximately 7% of participants meet the score cutoff for depression (4% “major depressive episode”, 1% “probably major depressive episode”, and 2% “possible major depressive episode”). However, due to the removal of the suicidal ideation item, this may under-identify the number of participants meeting CESD-R cutoff score criteria for each category. 

### 2.3. Procedure

This study was approved by the IRB at the institution of the first author. Participant responses were collected anonymously through an online survey tool. Students were offered entry into a raffle for a gift card for participation.

### 2.4. Data Analysis

The sample was randomly divided into non-overlapping exploratory (EFA, *n* = 197) and confirmatory (CFA, *n* = 201) subsamples. EFA was conducted using principal-axis factoring extraction. Promax rotation, an oblique rotation, was used because of the suspected intercorrelation between the factors and its ability to better identify a simple structure compared to orthogonal rotations [42,43]. A bifactor structure was examined using the Schmid–Leiman (1957) approach [44] in the MacOrtho program [45]. Reliability estimates were calculated using Omega hierarchical reliability [46,47]. Subsequently, CFA examined one-factor, three-factor, and three-factor bifactor models using WLSMV estimation [48,49]. Criteria for evaluating an acceptable model fit were established a priori: RMSEA values ≤ 0.08 and CFA values ≥ 0.90 [50,51,52].

## 3. Results

### 3.1. Preliminary Data Analysis

Missing data were minimal (<3%), and listwise deletion was used [53,54]. Univariate descriptive statistics are reported in Table 1.

### 3.2. EFA

EFA results (scree plot, parallel analysis, and MAP) supported two-and three-factor solutions. The two-factor solution accounted for 53% of the variance, and communalities ranged from 0.33 to 0.74. The three-factor solution accounted for 57% of the variance, and communalities ranged from 0.37 to 0.76. The three factors (Negative Affect (α = 0.93), Positive Affect (α = 0.89), Daily Functioning (α = 0.76)) have moderate factor inter-correlations (0.510 to 0.681; see Table 2), suggesting a bifactor structure [55]. Hence, a bifactor solution with one general factor and three subscale factors was examined. The Schmid and Leiman [44] analysis revealed that the general factor accounted for 39% of the total variance and 71.7% of the common variance. The combination of the general and specific factors accounted for 54.8% of the variance in the TDI. The Omega coefficients were high for the general factor (ω = 0.953), as were the subscales Negative Affect (ωs = 0.926), Positive Affect (ωs = 0.896), and Daily Functioning (ωs = 0.787). The Omega hierarchical coefficient for the general factor is 0.833, compared to the Omega coefficient of the general factor of 0.953; this indicates that most of the total score variance is attributable to the general factor. Thus, an interpretation of scores should fall primarily at the general factor level. However, some degree of interpretation at the subscale level is also appropriate.

### 3.3. CFA

**Figure 1:**
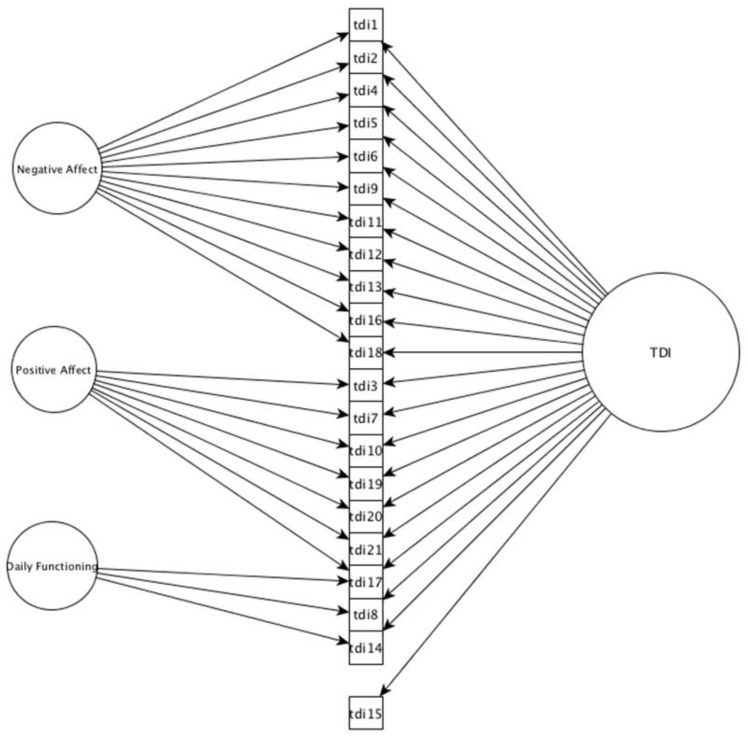
Bifactor model for the Teate Depression Inventory. *Note.* The path between item 15 and Factor II (Positive Affect) was dropped due to non-significant loading (*p* = 0.728) after the addition of the general factor. Correlated residuals and standard error are not depicted for readability. TDI—Teate Depression Inventory.

Previous research suggested a unidimensional TDI structure [18], while EFA findings from this study support a three-factor, bifactor structure. Thus, one-factor, three-factor, and three-factor bifactor models were tested and compared (see Table 3). All tested models included design-driven correlated residuals [56,57]. The CFA results suggested that the one-factor model and three-factor model with correlated factors yielded inadequate fits (See Table 3). The three-factor bifactor model was supported by the data and had an adequate fit: CFI = 0.975, RMSEA = 0.075 (see Figure 1).

### 3.4. Concurrent Validity

Bivariate correlations were used to examine the concurrent validity of the TDI with the CESD-R. The total scores (r = 0.83, *p* < 0.001) and subscales (0.58 to 0.94, *p* < 0.001) of the two scales were significantly correlated (See Table 4).

## 4. Discussion

### 4.1. Psychometric Findings

The present study developed the TDI-English version and examined the psychometric properties of TDI scores among young adults in the United States. The findings support a three-factor bifactor structure that corresponds closely with the Tripartite Model and the concurrent validity of TDI scores among this non-clinical sample.

Prior research on the TDI in Italy supported a unidimensional structure [28,29]. The present findings, supporting a bifactor structure, are in some ways consistent with prior findings, given the strength of the identified general factor. However, with a United States sample, in addition to the general factor, three theoretically consistent lower-order factors emerged. Notably, this study was the first to examine the TDI in the United States and the first to examine the TDI in any setting using a factor analytic approach. Thus, it is unclear whether these discrepant findings are the result of cultural differences between the United States and Italy or methodological ones (factor analytic approach vs. Rasch modeling). However, these findings are important as they suggest that additional interpretation of TDI scores beyond the general factor may be useful in the United States. The results supported the concurrent validity of the TDI (general factor and subscales) with the widely used CESD-R. Overall, these findings provided support for the structural and convergent validity of both the general factor and the subscales.

### 4.2. The TDI and the Tripartite Model

The TDI factors corresponded well with the aspects of the Tripartite Model that are relevant to depression—PA and NA. Clark and Watson [22] posited that depression is often marked by low PA and high NA. Factor I (TDI—Positive Affect) and Factor II (TDI—Negative Affect) are consistent with Clark and Watson’s conceptualizations of these factors in their model. The TDI does not have a factor representing the third aspect of the Tripartite Model (PH). However, PH is primarily associated with anxiety, not depression, and is primarily comprised of somatic symptoms—which can complicate the assessment of psychological disorders (as discussed previously). The TDI is a narrow-band measure intended to assess depression, rather than anxiety; thus, the absence of a PH factor is not overly concerning. Factor III (TDI—Daily Functioning) does not directly reflect any aspect of the Tripartite Model. However, this factor is still important, as it may serve as an indicator of the impact of the depressive symptoms on daily functioning. Because diagnosis of depression depends on the extent to which symptoms impact daily functioning, Factor III (TDI—Daily Functioning) of the TDI may potentially be clinically important, although more research is needed to evaluate clinical relevance.

### 4.3. Implications: A Preliminary Step

Overall, these findings suggest that the TDI shows promise as an instrument that may be useful in assessing depression for young adults in non-clinical settings. While several well-established self-rating depression tools with strong psychometric properties are available in the United States, these measures may include somatic symptoms (e.g., PHQ-9, CESD-R) or omit questions on suicidal ideation (e.g., HADS). The TDI was designed to assess cognitive and affective symptoms (including suicidal ideation) without relying on somatic symptoms, which may complement existing instruments and offer clinicians and researchers an alternative brief self-report measure of depression.

This study is intended to be a preliminary, yet critical, first step in evaluating the psychometric properties of the TDI within the United States. Importantly, the TDI was originally developed to be a measure that functions well across cultures and that is well-suited for individuals with chronic health conditions. Whether the scale is appropriate for those purposes is yet to be determined. However, this study represents an important first step in that process by adapting and examining the scale in a different cultural setting and testing the validity with a non-clinical sample. At present, findings from this study affirm the validity of TDI scores among a non-clinical sample of young adults in the United Sates, and the measure is already well-supported among members of many different groups in Italy.

### 4.4. Strengths and Limitations

This study’s strengths include utilizing an intensive translation/back-translation method consistent with ITC guidelines [20], examining the TDI using a bifactor modeling approach that has been shown to be superior relative to hierarchical and other modeling approaches [55], and adapting the TDI in the U.S. to potentially facilitate cross-cultural research in the future.

As with all research, there are also several limitations. First, as this is one of the first psychometric studies examining the TDI, the generalizability of the findings may be limited. Participants in this study were from a single university. The English used in the university could differ from other English-speaking countries outside of the United States. Additionally, the sample used in this study was relatively small and did not include individuals with chronic health conditions. However, the potential use of the scale for individuals with chronic health conditions is an important part of the appeal of the TDI and a critical avenue for future research. Therefore, an ongoing psychometric investigation of the English version of the TDI across diverse samples will be essential.

### 4.5. Future Directions

Future research examining the generalizability of these findings—particularly to individuals with chronic health conditions and peripartum/postpartum women—would be particularly valuable. Given the de-emphasized role of somatic symptoms on the TDI, the scale has the potential to be particularly useful for these populations and for studies comparing depression across individuals with and without chronic health conditions, but further psychometric evidence, including evidence of measurement invariance, is needed. Future studies should also examine the psychometric characteristics of the TDI using both non-clinical and clinical samples in the United States to determine cutoff scores for symptom severity levels (minimal, mild, moderate, and severe depression) and to enhance the usefulness of the TDI. Moreover, while the TDI is not designed as a diagnostic instrument, research comparing the performance of the TDI to formal diagnostic tools and exploring the clinical utility of TDI scores (sensitivity, specificity) will be needed before the scale can be used to screen and measure symptom severity in clinical settings.

## 5. Conclusions

Depression is a global health concern, and the TDI is a newly developed self-report scale from Italy that measures depression. This study provided preliminary support for the adapted, English version of the TDI with a three bi-factor structure, which corresponds well to the Tripartite Model, indicating that this version of the TDI may be a useful measure in assessing depressive symptoms. Although future research is needed, the results of this study show that the TDI is a promising instrument for measuring depressive symptoms among a non-clinical population.

## Figures and Tables

**Table 1:** Descriptive statistics for 21 items from the Teate Depression Inventory.

Items	Variables	Mean	SD	Variance	Skewness	Kurtosis
1	Felt down	2.87	0.91	0.83	−0.05	−0.30
2	Difficulty concentrating	2.96	1.04	1.09	−0.07	−0.61
3	Worth living	1.94	1.21	1.47	1.18	0.35
4	Slow thinking	2.60	1.15	1.33	0.22	−0.79
5	Ashamed	2.26	1.46	1.10	0.52	−0.30
6	Felt withdrawn	2.26	1.21	1.46	0.56	−0.74
7	Felt proud	2.69	0.94	0.89	0.39	−0.23
8	Concentrated well	2.85	0.87	0.76	0.37	−0.20
9	Failure	2.16	1.13	1.27	0.70	−0.35
10	Enjoyment	2.09	0.94	0.89	0.70	0.23
11	Unable to accomplish goals	2.75	1.05	1.11	0.27	−0.35
12	Slow to complete tasks	2.57	1.08	1.16	0.30	−0.49
13	Unmotivated	3.18	0.98	0.96	−0.11	−0.33
14	Energy	2.73	0.99	0.97	0.36	−0.27
15	Happy	2.66	1.10	1.21	0.32	−0.57
16	Not enough energy	2.80	1.03	1.07	0.23	−0.42
17	Decisiveness	2.65	0.99	0.98	0.40	−0.07
18	Lost interest	2.25	1.11	1.22	0.73	−0.19
19	Felt had worth	2.21	1.10	1.22	0.69	−0.21
20	Enjoyed things	2.32 (*0.98*)	0.98	0.96	0.39	−0.35
21	Meaningful life	2.00 (*1.11*)	1.11	1.24	0.94	0.02

*Note*. *n* = 409. SD—standard deviation.

**Table 2:** Pattern coefficients for the three-factor model.

TDI Items		Pattern Coefficients		
		Negative Affect	Positive Affect	Daily Function
6	Felt withdrawn	**0.83**	0.17	−0.18
9	Failure	**0.82**	0.21	−0.19
5	Ashamed	**0.80**	−0.05	−0.06
11	Unable to accomplish goals	**0.78**	−0.10	0.06
1	Felt down	**0.72**	−0.01	0.01
2	Difficulty concentrating	**0.69**	−0.28	0.34
13	Unmotivated	**0.68**	−0.04	0.02
18	Lost interest	**0.65**	0.19	−0.16
16	Not enough energy	**0.61**	−0.16	0.36
12	Slow to complete tasks	**0.58**	−0.11	0.33
4	Slow thinking	**0.54**	−0.06	0.29
19	Felt had worth	0.18	**0.76**	−0.08
10	Enjoyment	−0.25	**0.74**	0.07
21	Meaningful life	0.22	**0.72**	0.01
3	Worth living	−0.15	**0.70**	−0.05
7	Felt proud	0.04	**0.55**	0.21
15	Happy	0.13	**0.55**	0.30
20	Enjoyed things	0.15	**0.49**	0.31
8	Concentrated well	−0.01	0.01	**0.87**
14	Energy	0.03	0.24	**0.55**
17	Decisiveness	−0.04	0.23	**0.54**

*Note*. *n* = 203. Pattern coefficients > 0.40 are boldface. TDI—Teate Depression Inventory. Items are abbreviated.

**Table 3:** Model-fit indices for the Teate Depression Inventory from confirmatory factor analysis.

Model	Index					
	χ_M_^2^	*df*_M_	*p*	RMSEA (90% CI)	CFI	TLI
One factor	764.809	177	<0.001	0.127 (0.118–0.136)	0.920	0.905
Correlated three-factor	2006.362	166	<0.001	0.232 (0.223–0.241)	0.748	0.682
Three factor Bifactor	338.075	156	<0.001	0.075 (0.064–0.086)	0.975	0.966

*Note.* χ_M_^2^—chi-square; *df*_M_—degrees of freedom; RMSEA—root-mean-square error of approximation; CI—confidence interval; CFI—Comparative Fit Index; TLI—Tucker–Lewis Index.

**Table 4:** Pearson correlation coefficients.

	TDI-NA	TDI-PA	TDI-DF	TDI-Total	CESD-R-NM	CESD-R-FI	CESD-R-Total
TDI-NA	1.00						
TDI-PA	0.63 **	1.00					
TDI-DF	0.76 **	0.73 **	1.00				
TDI-Total	0.94 **	0.85 **	0.87 **	1.00			
CESD-R-NM	0.78 **	0.68 **	0.63 **	0.81 **	1.00		
CESD-R-FI	0.80 **	0.58 **	0.69 **	0.79 **	0.80 **	1.00	
CESD-R-Total	0.83 **	0.65 **	0.70 **	0.83 **	0.92 **	0.96 **	1.00
*M*	28.72	15.91	7.64	52.27	5.78	12.62	17.60
*SD*	9.02	5.73	2.35	15.39	6.89	10.24	15.85

*Note. n* = 409. TDI—Teate Depression Inventory; TDI-NA—Negative Affect; TDI-PA—TDI-Positive Affect; TDI-DF—TDI-Daily Functioning; CEAD-R—The Center for Epidemiologic Studies Depression Sale-Revised; CESD-R-NM—Negative Mood; CESD-R-FI—CESD-R-Functional Impairment. ** *p* > 0.001.

## Data Availability

The data that support the findings of this study are available on request from the corresponding author [L.R.-I.]. The data are not publicly available due to information on the dataset that could compromise the privacy of the research participants.
